# Cogan’s syndrome is more than just keratitis: a case-based literature review

**DOI:** 10.1186/s12886-023-02966-6

**Published:** 2023-05-12

**Authors:** Yanqing Wang, Shichao Tang, Chong Shao, Yu Liu

**Affiliations:** Department of Rheumatology and Immunology, Tongren Hospital, Shanghai JiaoTong University School of Medicine, No. 1111 Xianxia Road, Changning District, Shanghai, 200336 China

**Keywords:** Interstitial keratitis (IK), Autoimmune inner ear disease (AIED), Cogan’s syndrome, biological agents, Tofacitinib

## Abstract

**Background:**

Cogan's syndrome (CS) is a rare autoimmune disorder characterized by non-syphilitic interstitial keratitis (IK) and Menière-like cochlear vestibular symptoms, which may also have systemic effects. Corticosteroids are first-line treatment. DMARDs and biologics have been used to treat ocular and systemic symptoms of CS.

**Case presentation:**

This is a case of a 35-year-old female who reported hearing loss, eye redness and photophobia. Her condition progressed to a sudden sensorineural hearing loss, tinnitus, and constant vertigo accompanied by cephalea. CS was diagnosed after excluding other diseases. The patient still developed bilateral sensorineural hearing loss after receiving hormone, methotrexate, cyclophosphamide, and a variety of biological agents. Joint symptoms were relieved after treatment with a JAK inhibitor (tofacitinib), and hearing did not deteriorate further.

**Conclusions:**

CS should be involved in the differential diagnosis of keratitis. Early identification and intervention of this autoimmune disease can minimize disability and irreversible damage.

## Background

Cogan’s syndrome (CS) is a rare systemic vasculitis that can severely affect vision and hearing and may also have significant systemic effects [1]. CS primarily affects young Caucasian adults in their 20 s and 30 s, with no significant differences by gender or race [2–4]. The most common ophthalmic symptom of CS is interstitial keratitis (IK). This can manifest as redness of the eyes, sensitivity to light, eye irritation, and blurred vision. The most common causes of IK are contagious. Ocular manifestations of CS tend to respond well to corticosteroids and disease-modifying anti-rheumatic drugs (DMARDs) [5]. However, clinicians often do not include CS in the differential diagnosis of patients with IK, resulting in delayed or recurrent disease. Blindness has been reported in 8% of patients [6].

Vestibular auditory dysfunction in CS with sudden onset of Menière-like vertigo, ataxia, tinnitus, nausea, vomiting, and sudden hearing loss [6]. Testing for sensorineural hearing loss includes audiometric assays that typically affect both low and high range frequencies. Computed tomography (CT) scans and magnetic resonance imaging (MRI) may be normal. Nearly 43% of CS patients develop deafness, which is usually irreversible. Most untreated patients had moderate, or severe hearing loss during the 5-year follow-up period. However, early treatment (within 2 weeks of initiation) with high-dose oral corticosteroids can reduce the severity of hearing loss to moderate or mild, but not completely reverse the loss [7, 8].

CS has been classified as a primary variable vessel vasculitis. Approximately 80% of patients have features of various systemic manifestations, including fever, arthralgia, anemia, neurological and gastrointestinal disorders [9]. Aortitis is the most common systemic vasculitis that presents in approximately 10% of CS patients [10]. Involvement of the branches of the aorta may result in claudication of the upper and lower limb [11, 12].

The diagnosis of CS can be elusive due to the variability of clinical presentations. The diagnosis of typical CS is based on non-syphilitic IK with acute vestibular symptoms within 2 years [13]. Since these symptoms are often presented to different specialists, there may be a delay in linking them. If not treated promptly, the prognosis is likely to be poor. Therefore, this report aims to familiarize clinicians with the clinical manifestations and the latest medical management of CS, in order to provide patients with timely and effective treatment.

## Case presentation

A 35-year-old female presented with fever, diarrhea, headache, bilateral sensorineural hearing loss, and tinnitus to the emergency department. She first developed earplugs and tinnitus in November 2009, which eased after rest. In the same year, sudden hearing loss (50 decibels, high frequency) occurred in the left ear, accompanied by dizziness, nausea, and vomiting. Oral corticosteroids and hyperbaric oxygen therapy (HOT) improved her symptoms. In February 2011, she first presented with ocular symptoms, including swelling and redness in her right eye. One year later, she developed blurred vision, mainly in her right eye. Oral and topical corticosteroids relieved her symptoms but recurred when she stopped using them. In 2016, she suffered from finger and knee pain, vomiting, and diarrhea. There was no family history of vascular disease or collagen disease.

**Fig. 1:**
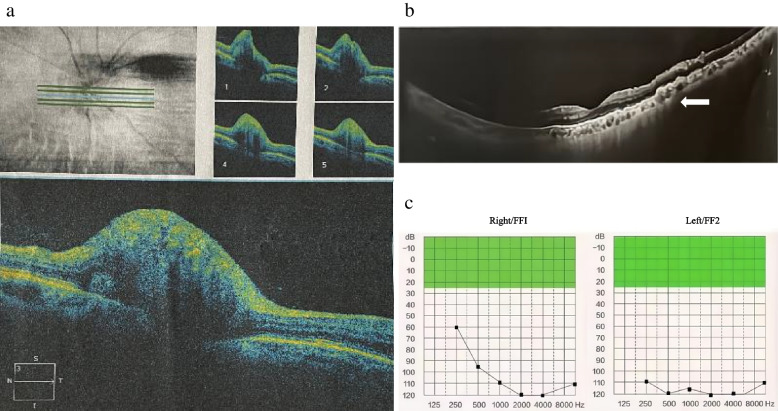
**a** OCT: optic disc oedema **b** OCT: macular degeneration **c** Pure tone audiogram: bilateral severe sensorineural hearing loss

Clinical examination revealed bilateral red eyes, swollen eyelids, lacrimation, and ocular secretions. An ophthalmological examination was performed: Intraocular pressure was normal. Slit lamp examination showed signs of keratitis. Orbital ultrasonography revealed a thickening of the medial rectus muscle. Optical coherence tomography (OCT) showed signs of optic disc oedema and macular degeneration. Fundus angiography showed no abnormalities. An audiogram revealed a pattern of sensorineural hearing loss. The degree of hearing loss was extremely severe in both ears (Fig. 1). CT and contrast-enhanced MRI of the inner ear were normal. However, brain MRI showed patchy hyperintensity in the centrum semivale.

Laboratory tests showed elevated erythrocyte sedimentation rate (ESR) and C-reactive protein (CRP) (ESR, 69 mm/h; CRP, 151 mg/L). The white blood cell count was 14,290/μL (84.5% neutrophils) and the interleukin-6 titer was 25.9 pg/mL. Anti-nuclear antibody (ANA), myeloperoxidase anti-neutrophil cytoplasmic antibody (MPO-ANCA), protease 3-ANCA, anti-cyclic citrullinated peptide antibody, anti-dsDNA antibody, anti-extracted nuclear antigen peptide antibody (anti-ENA antibody) and rheumatoid factor, liver and kidney function tests were normal. Treponema pallidum antibodies and cultured cerebrospinal fluid (CSF) were negative. Her electrocardiogram, transthoracic echocardiogram, and CT of the chest were normal. The diagnosis of Cogan syndrome was based on a combination of bilateral keratitis, sensorineural hearing loss, and systemic vasculitis manifestations.

The patient initially received intravenous methylprednisolone 40 mg/day in combination with oral methotrexate 10 mg/week. Ocular symptoms and hearing were relieved to normal levels. Symptoms recur during corticosteroid tapering. In October 2016, she suffered multiple joint swelling and pain, severe hearing loss in both ears (left ear 120 decibels, right ear 80 decibels) with elevated inflammatory indicators (CRP, 69 mg/L; ESR, 108 mm/h). Methylprednisolone (40 mg/day), methotrexate (10 mg/week), and cyclophosphamide (0.4 g/week) were prescribed to improve her symptoms. One month later, her inflammatory indicators decreased, but her hearing and joint symptoms did not ease. She was prescribed with rituximab 500 mg/week for 3 months. Her tinnitus, nausea and vomiting improved significantly. Unfortunately, her hearing function showed little improvement. In April 2017, she had a fever with diarrhea, visual field defect, headache, tinnitus, and increased blood interleukin-6 levels. The interleukin-6 inhibitor tocilizumab (TCZ) (480 mg/month subcutaneously) was then added. Her inflammatory markers level normalized immediately (CRP, 0.6 mg/L; ESR, 3 mm/h). Although the audiometry results did not improve significantly, she regained the ability to hear loud conversations. Ocular lesions did not progress. Although we increased the dose of MTX to 15 mg/week, a relapse occurred when the prednisolone dose was reduced to 20 mg/day. The patient developed glucocorticoid side effects in January 2019. The dose of glucocorticoids was gradually reduced and a tumor necrosis factor (TNF)-α inhibitor (etanercept 25 mg, 2 times/week) was administered. Methotrexate therapy continued with hydroxychloroquine adjuvant therapy. The incidence of conjunctivitis and eyelid edema decreased, and hearing did not deteriorate further. During hospitalization, the patient complained of fever, cough, nausea, vomiting, dizziness, and tinnitus. CRP (151.79 mg/L) and ESR (69 mm/h) were elevated. She was prescribed with methylprednisolone (60 mg/d), TNF-α inhibitor (etanercept 25 mg, 2 times/week), and immunoglobulin (2.5 g/d × 3d). The patient's body temperature gradually returned to normal. Respiratory and tinnitus, dizziness symptoms eased, and inflammatory markers decreased (CRP, 39 mg/L; ESR, 29 mm/h). After discharge, the patient was followed up regularly in the outpatient clinic. During the gradual tapering of hormones, to relieve the symptoms of joint pain and tinnitus, we tried to treat her with a Janus kinase (JAK) inhibitor (tofacitinib, 10 mg/day), and the patient's inflammatory indexes returned to normal. As maintenance therapy, prednisone was reduced to 5 mg/day orally. Symptoms such as fatigue, anxiety and joint pain have been relieved. There were no serious infections other than occasional mild cough and diarrhoea.

## Discussion and conclusions

The etiology of CS remains incompletely understood. There is evidence that CS may be an autoimmune disease, or even vasculitis, based on antibodies against inner ear and corneal tissue and evidence of cell-mediated immunity [14, 15]. Since CS is a rare disease, only a few hundred cases have been reported in the literature since 1945. To date, the diagnosis of CS has been based on clinical findings in the absence of specific tests (Table 1) [4, 16–26].

**Table 1:**
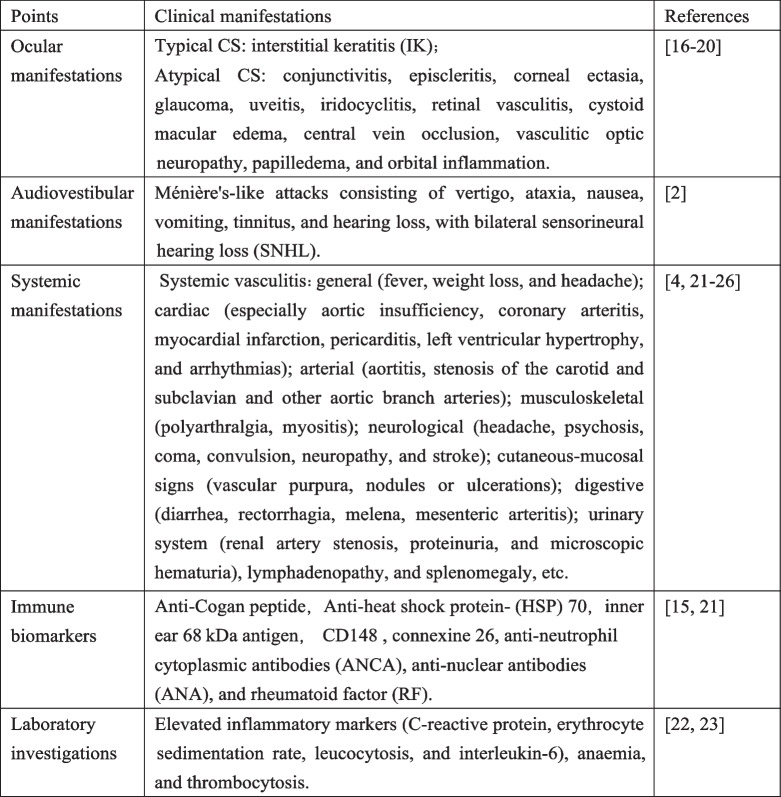
Clinical manisfestation of Cogan’s syndrome

Suspicion of CS arises when a patient presents with ocular inflammation (especially IK) with or without symptoms of hearing loss. However, other infectious or inflammatory causes of ocular symptoms or vestibular symptoms need to be excluded (Table 2) [27–32].

**Table 2:**
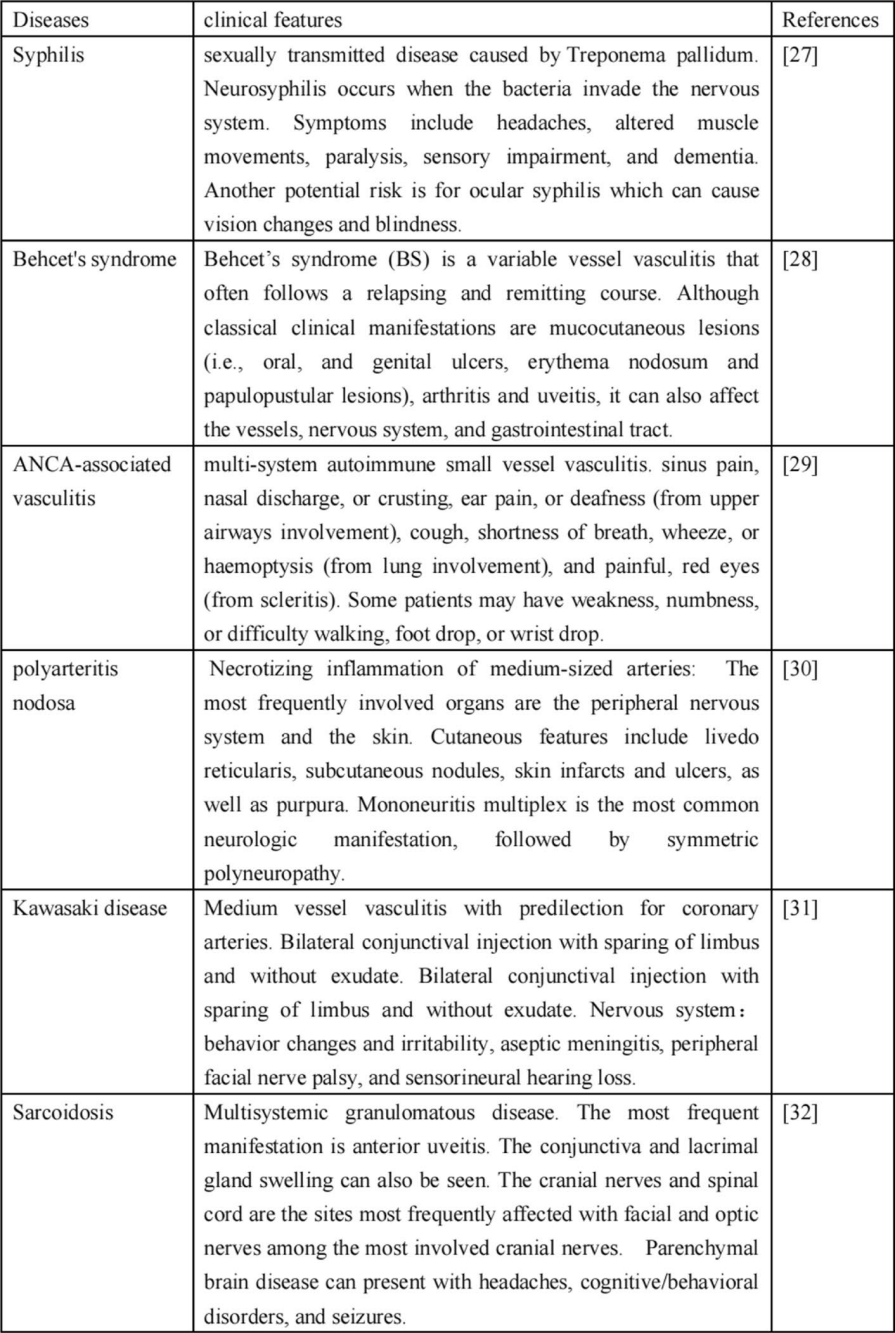
Differential diagnosis for Cogan’s syndrome

Once a diagnosis of CS has been made, patients need to be evaluated for vasculitis in addition to the degree of visual and hearing loss. 10%-15% of CS patients may have large and/or medium vessel vasculitis. 10% of CS patients reported that aortic root vasculitis may lead to life-threatening complications such as aortic aneurysm, renal artery stenosis, splenic aneurysm, superior mesenteric aneurysm, coronary artery stenosis, cerebral hemangioma and stenosis, and heart valve disease. Therefore, CS requires the multidisciplinary participation of ophthalmologists, otolaryngologists, rheumatologists, and surgeons in clinical practice. In addition to perfecting eye exams and pure tone audiograms for quantifying hearing loss and tracking disease progression, laboratory tests (including complete blood count, urinalysis, serum electrolytes, creatinine, hepatic transaminases, and erythrocyte sedimentation rate) are also required to confirm systemic involvement or other diagnoses [33]. Simultaneous echocardiography is necessary to evaluate for evidence of aortitis with aortic valvular dysfunction. Coronary angiography is also required if there are signs or symptoms of ischemic heart disease. When vasculitis at other sites is suspected, it should be recommended to complete MRA, angiography, and even positron emission tomography combined with computed tomography (PET-CT) [34, 35].

At present, there is no clear cure for CS. Corticosteroids are the cornerstone of CS treatment during flare-ups and in the acute phase of disease. Traditional immunosuppressive therapy, such as Methotrexate, can be used in relapsed patients or as a glucocorticoid sparing agent. TNF-α blockers and other biologics add promising options for the management of severe and/or refractory forms [33].

### Corticosteroids

Glucocorticoids are the first-line treatment for CS and are usually effective for controlling ocular, vascular, or other visceral symptoms, but less reliable for hearing. It is recommended to start with 1 mg/kg/day of prednisone for 2–4 weeks or until patient improves [23]. Hearing loss recovery may be more likely when corticosteroids are given early in the course of the disease. Although successful responses can be achieved, relapses are common due to long-term glucocorticoid use, and side effects can occur. Treatment failure indicates the need to add additional immunosuppressants [4].

### Methotrexate

Open-label studies have shown that methotrexate may be beneficial in some patients, especially for those with mild disease, but controlled randomized trials did not support its use [36, 37].

### Cyclophosphamide

Treatment with cyclophosphamide has been proposed to improve outcomes in autoimmune inner ear disease [38]. Although some success has been reported in slowing or preventing hearing loss, cyclophosphamide has been reported to be associated with significant toxicity, including an increased risk of infection, malignancy, and death.

### Rituximab

Rituximab is a genetically engineered, chimeric murine/human monoclonal antibody directed against the cluster of differentiation (CD)-20 antigen found on the surface of normal and premalignant B cells and mature B cells. In CS, hearing function appears to be the most difficult parameter to control. Therefore, the effects of rituximab on B cells may help avoid deafness and the need for cochlear implants in severe cases. It can also significantly reduce the amount of medication needed to control multiple manifestations of CS [39].

### Tocilizumab

Tocilizumab (TCZ) is a recombinant humanized monoclonal anti-interleukin 6 (IL-6) receptor antibody that inhibits both membrane-bound and soluble IL-6 receptors [40]. It has been reported in the literature that the clinical symptoms of patients with long-term drug-resistant CS were significantly improved by intravenous administration of TCZ at a dose of 8 mg/kg [41].

### Etanercept

Etanercept is a fusion protein consisting of 2 recombinant p75 TNF receptors linked to the Fc portion of human IgG. It is a powerful antagonist of TNF, binding to and inactivating the cytokine. In an open-label prospective pilot study, 23 patients with bilateral immune-mediated cochlear vestibular disease or symptoms of bilateral Meniere's disease received etanercept (25 mg twice weekly, subcutaneous injection) for 24 weeks. Hearing improved in 7 (30%) patients, unchanged in 13 (57%) and worsened in 3 (13%) patients [42].

### Tofacitinib

Tofacitinib is a potent, selective Janus kinase (JAK) inhibitor that preferentially inhibits JAK1 and JAK3. Zhang et al found that tofacitinib potently inhibits tissue-resident memory T cells and inhibits core angiogenic effector pathways, thereby suppressing pathogenic immune responses in medium and large vasculitis [43]. A pilot study shows that tofacitinib is well tolerated and effective for patients with non-organ-threatening ANCA-associated vasculitis [44].

### Infliximab

Infliximab is a monoclonal antibody designed to intercept and neutralize the key inflammatory cytokine TNF-α. Infliximab is emerging as a potential first-line therapy in combination with corticosteroids. Durtette et al. reported an 80% response rate to vestibular auditory symptoms with infliximab in patients who had failed multiple corticosteroids and DMARDs [5].

### Certolizumab pegol

Certolizumab pegol (CZP) is a particularly attractive second line therapy to avoid inflammatory flares and thereby protect the mother and her unborn child. The European League Against Rheumatism (EULAR) recommends certolizumab pegol as the most favorable biological DMARD throughout pregnancy and lactation in patients with inflammatory rheumatic diseases [45]. A report of three pregnancies in two patients showed that CZP was an effective and safe treatment of CS [46].

### Plasmapheresis

Plasma exchange is frequently used for neuroimmune diseases such as multiple sclerosis, acute and chronic inflammatory demyelinating neuropathy, or refractory myasthenia gravis. Plasma exchange is increasingly used in the treatment of vasculitis [29], and should be considered in patients with chronic inflammatory activity and resistance against or severe side effects to other therapeutic concepts [47].

### Otologic surgical interventions

Cochlear implantation (CI) is a highly effective way of hearing rehabilitation. CI in CS patients can be technically challenging due to partial occlusion or complete neo-ossification of the intracochlear duct due to an inflammatory endosteal response [48]. Skin atrophy from long-term corticosteroid and immunosuppressive therapy and ischemia from vasculitis may be risk factors for complications of wound healing [49]. However, data from two long-term follow-up studies suggest that CS patients gain significant open-set speech recognition benefits from long-term stable CI [50].

In summary, CS should be part of the differential diagnosis of keratitis, especially in the absence of predisposing factors to infection. It is important to carefully assess whether the patient has vestibular disturbances such as dizziness, ataxia, tinnitus, nausea, vomiting and sudden hearing loss. Simultaneously, it is necessary to carefully inquire if the patient has systemic symptoms such as fever, arthralgia, anaemia, neurological, gastrointestinal or limb claudication. Corticosteroids and DMARDs remain the first line treatment for CS, while biological agents and JAK inhibitors are considered to be promising options for severe and/or refractory patients.

## Data Availability

The datasets used and analyzed during the current study are available from the corresponding author on reasonable request.

## Abbreviations

CS: Cogan’s syndrome
IK: Interstitial keratitis
AIED: Autoimmune inner ear disease
DMARDs: Disease-modifying anti-rheumatic drugs
CT: Computed tomography
MRI: Magnetic resonance imaging
HOT: Hyperbaric oxygen therapy
OCT: Optical coherence tomography
ESR: Erythrocyte sedimentation rate
CRP: C-reactive protein
ANA: Antinuclear antibodies
ANCA: Anti-neutrophil cytoplasmic antibody
SNHL: Sensorineural hearing loss
RF: Rheumatoid factor
TNF: Tumor necrosis factor
